# Physical Activity, Cognitive Function, and Brain Health: What Is the Role of Exercise Training in the Prevention of Dementia?

**DOI:** 10.3390/brainsci2040684

**Published:** 2012-11-29

**Authors:** Sara M. Gregory, Beth Parker, Paul D. Thompson

**Affiliations:** Department of Preventive Cardiology, Hartford Hospital, 80 Seymour Street, Hartford, CT 06102, USA; Email: bparker03@harthosp.org (B.P.); pthomps@harthosp.org (P.D.T.)

**Keywords:** cognitive function, hippocampus, exercise, fitness, dementia

## Abstract

The population of elderly adults in the US is growing, and the prevalence of age-related cognitive decline and dementia is expected to increase in turn. Effective and inexpensive interventions or preventive measures are necessary to attenuate the increased economic and social burden of dementia. This review will focus on the potential for physical activity and exercise training to promote brain health and improve cognitive function via neurophysiological changes. We will review pertinent animal and human research examining the effects of physical activity on cognitive function and neurophysiology. We will discuss cross-sectional and longitudinal studies addressing the relationship between neurocognitive health and cardiorespiratory fitness or habitual activity level. We will then present and discuss longitudinal investigations examining the effects of exercise training on cognitive function and neurophysiology. We will conclude by summarizing our current understanding of the relationship between physical activity and brain health, and present areas for future research given the current gaps in our understanding of this issue.

## 1. Introduction

**Figure 1:**
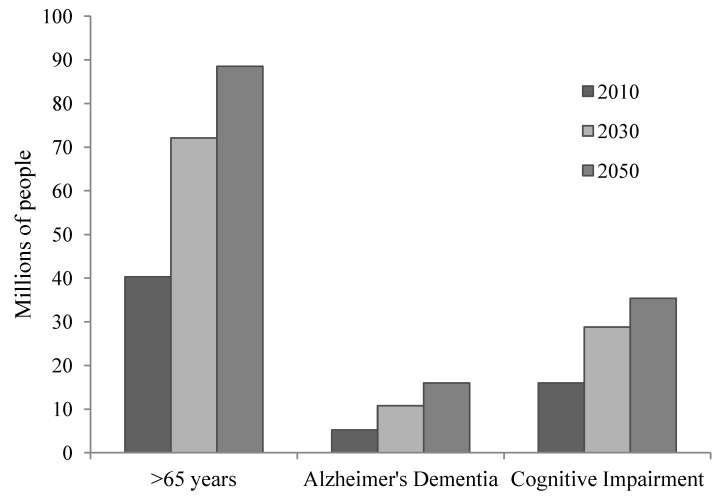
Prevalence (2010) and projected population estimates in 2030 and 2050 for the number of US adults over age 65, with Alzheimer’s dementia, and with cognitive impairment based on US Census Bureau population projections.

The elderly population in the United States is expected to increase dramatically by mid-century. US Census Bureau projections estimate that the population of US residents over age 65 will more than double between 2008 and 2050, and 1 in 5 US residents will be over the age of 65 by 2030 [1,2] (Figure 1). A progressively older population increases the social and economic burdens required to care for the physiological consequences of the aging process, including the structural and functional changes in the brain associated with a decline in cognitive function [3]. Age-related cognitive changes may progress from mild cognitive impairment (MCI) to dementia—a condition in which memory, behavior, and cognition are impaired secondary to neurodegeneration in the brain. Alzheimer’s disease (AD) is diagnosed when these neurodegenerative brain changes prevent an individual from performing basic physical tasks and bodily functions [4]. It is estimated that 5.4 million people suffer from AD in 2012 (5.2 million over the age of 65) and healthcare costs related to AD are estimated at between $130 and $200 billion in the US [4,5]. These costs are projected to reach $1.1 trillion by 2050. As the elderly population grows, the healthcare-related financial burden will increase, and the need for pharmacological and non-pharmacological prevention and treatment for these conditions increases. This review of literature will discuss the potential for regular physical activity to maintain cognitive function and normal neurophysiology, and prevent the progression from mild cognitive impairment to dementia. 

## 2. Aging and Brain Integrity

Normal aging is associated with structural and neurophysiological changes in the brain as well as a decline in cognitive function. Age has been correlated with a loss of cerebral cortical tissue, most consistently in the frontal cortex and hippocampus. Age has also correlated with a loss of white matter in the frontal, occipital, temporal, and parietal regions and cerebellum [6]. A longitudinal analysis of adults age 64 to 86 (120 normal and 18 with MCI) showed a decline over 10 years in brain volume of all regions scanned by magnetic resonance imaging (MRI) [3]. Some areas were reported to show accelerated decline with aging, which included the ventricular cerebrospinal fluid (CSF), frontal gray matter, and areas of the frontal and parietal lobes. Volume losses in the ventricular CSF, gray matter, hippocampus, orbitofrontal, middle temporal, and perirhinal cortices were greater over 10 years in subjects with MCI [3]. Hippocampal volume loss in elderly subjects may be related to cognitive impairments. Elderly subjects (age 60–85) demonstrated impaired memory task performance, smaller hippocampal volumes, and lower *n*-acetylaspartate/creatine (NAA/Cr) ratios in the frontal white matter and hippocampus when compared to young subjects (age 20–39) [7]. Hippocampal volume and NAA/Cr ratio correlated with performance on cognitive tasks in elderly subjects [7]. NAA is a brain-specific metabolite involved in myelin turnover and the NAA/Cr ratio is considered an indicator of neuronal health with lower ratios indicating neurodegeneration [8]. Thus, brain tissue atrophy and neurophysiological changes occur with aging and may be related to changes in cognitive function. 

The age-related loss of brain tissue and neurophysiological changes appears to be region-specific. The hippocampus has been studied intensively because of its role in facilitating memory-related tasks. Blood flow measured by positron emission tomography (PET) increased to the medial temporal lobe, which includes the hippocampus, during episodic memory recall in middle-aged subjects [9], and hippocampal volume was associated with performance on tests of memory-related cognitive function [7,10]. The functional importance of the hippocampus is evidenced by reports of impaired performance on spatial memory tasks in rats with hippocampal lesions and in humans with hippocampal resections [11,12]. Furthermore, a 9-year, prospective study demonstrated that greater gray matter volume in the hippocampus, frontal gyrus, and supplementary motor area was associated with a lower risk of developing MCI [13]. Hippocampal atrophy, which occurs normally with aging, may be accelerated in those who progress from mild cognitive impairment (MCI) to dementia [10,14]. These findings suggest that interventions aimed at preventing hippocampal atrophy and neurodegeneration may prevent age-related cognitive/memory impairments and associated structural and function brain changes. 

## 3. Exercise Training and Cognitive Function

### 3.1. Animal Studies

Several animal models of physical activity and exercise training have demonstrated beneficial effects on cognitive function. Daily wheel running for 2 weeks [15] or increased physical exercise for 7 weeks [16] improved memory and learning performance in mice, although cognitive stimulation was also provided to the animals that had exercised in the latter study. A combination of physical and cognitive activities improved memory performance in rats when compared to either physical or cognitive activity alone and sedentary controls [17]. Furthermore, aerobically-trained middle-age and old mice performed better on tasks involving memory and learning than sedentary controls [18]. Similar improvements in memory and learning occurred after 5 days of wheel running [19] and 4 weeks of treadmill running [20] in mice. 

In addition to cognitive improvement, animal models also report protective effects of exercise on cognitive function. Age-related impairments in memory and spatial learning were reversed by increased physical activity in older mice [21,22]. Similarly, chronic aerobic exercise maintained cognitive function in mice exposed to stress induced by cortisone administration or immobilization [21,22,23]. These collective findings indicate that physical activity may improve or reverse age- and stress-related impairment in hippocampal-related cognitive tasks (*i.e.*, memory and learning), and provide the framework for the human studies that will be discussed below exploring the relationship between physical activity and cognitive impairment. 

### 3.2. Human Studies

#### 3.2.1. Cross-Sectional and Observational Investigations

Cross-sectional studies in humans suggest that more active individuals may have reduced risk of cognitive impairment and dementia [24,25,26,27,28]. Individuals that reported greater amounts of light exercise had a reduced odds ratio for all-cause and Alzheimer’s dementia when compared to those reporting no activity [29]. Cognitive impairment was more prevalent in those reporting no activity *versus* moderate and high activity levels in a community-based study of persons over 55 years [30]. Moreover, higher self-reported activity levels at mid- and late-life were associated with a reduced odd ratio for MCI in late life, and physical activity levels at various points across the lifespan (teenage, age 30, age 50, and age >65) were associated with reduced risk of cognitive impairment in older adults [25,31]. Self-reported history of high-intensity exercise was associated with better cognitive performance in those over 80 years, but worse cognitive performance in postmenopausal women [32,33]. Moderate activity levels, however, were correlated with better cognitive performance in postmenopausal women [33]. Older (>60 years) marathon runners and bicyclists performed better than inactive controls on only one cognitive task—the Five Point Test—of the Vienna Neurophysiological and Cerad Test Batteries [34]. A meta-analysis of 37 studies, including cross-sectional and longitudinal studies, concluded that increased physical activity was associated with better cognitive performance, although the average effect size was small and there was a wide range of effect sizes for individual studies (for example −1.08 to 2.56 for the relationship between physical activity and cognition in cross-sectional designs). These collective findings indicate that increased physical activity may improve cognition and/or reduce the likelihood of cognitive decline and dementia, but the effect may be small and variable. 

Prospective longitudinal investigations have attempted to determine the effects of physical activity or fitness on cognitive decline or incident dementia over follow-up periods of several years (Table 1). Higher physical activity levels at baseline were associated with less cognitive decline over 2- to 8-year follow-up periods [25,26,27,28,30,35,36,37,38,39]. In a large prospective investigation, individuals in the middle and highest tertiles of cardiorespiratory fitness had a reduced risk for dementia-related mortality over an average follow-up period of 17 years when compared to the lowest tertile [26]. The most significant reduction in risk was seen between the lowest and middle fitness tertiles, and each 1-metabolic equivalent (MET) improvement in fitness reduced the relative risk of dementia-related mortality by 14%. Similarly, a meta-analysis of 16 prospective studies reported a reduction in relative risk for dementia in the highest physical activity category when compared to the lowest [40]. Comparison of cross-sectional and prospective analyses is made difficult by differences in the method used to measure physical activity level: questionnaire [27,28,35,39], self-reported activities [28,30,37], and more objective measures, including active energy expenditure [38], 24-h actigraphy [24], or cardiorespiratory fitness [26]. In addition, different activity status classifications have been used, such as dichotomizing the sample into active and inactive subjects based on arbitrary cut points [35,37,38], differentiating between levels of activity (*i.e.*, low, moderate, and high) [30], or establishing percentiles [28,38,39]. Studies also differ in the outcome variable measured (*i.e.*, cognitive performance, dementia development, Alzheimer’s risk or mortality) and measurement technique (*i.e.*, cognitive test battery used, criteria for dementia or cognitive impairment). Such differences make it difficult to compare studies and draw firm conclusions. 

**Table 1:** Summary of cross-sectional and observational studies regarding physical activity, fitness, and brain function.

Study	*N*	Fitness/Activity Indicator	Primary Outcome Variable	Primary Finding
Erickson *et al.* 2010 [13]	299	Physical activity level measured at baseline as blocks walked per week	Gray matter volume (GMV) at 9-year follow-up visit	Walking distance predicted GMV 9 years later. Areas included frontal, parietal, and occipital lobes, entorhinal cortex, and hippocampus.
Buchman *et al.* 2012 [24]	716	Total daily physical activity (PA) based on 24-h actigraphy for 10 days	Incidence of Alzheimer’s dementia and cognitive decline (performance on battery of 19 cognitive tests) over 4 years	Daily PA was associated with the risk of developing AD ^1^ based on Cox proportional hazards (individual in 10th percentile had >2-fold greater risk of AD than person in 90th), and was associated with the level and annual rate of decline in global cognitive function.
Geda *et al.* 2010 [25]	1324	Physical activity (determined by questionnaire) in mid- life (age 50 to 65) or late life (age 70-89)	Odds of developing MCI ^2^ in later life	The odds ratio for development of MCI was lower for any frequency of moderate intensity physical activity performed in mid-life (OR = 0.61) and late-life (OR = 0.68).
Liu *et al.* 2012 [26]	14,811 women and 45,078 men (age 20 to 88)	Cardiorespiratory fitness (CRF)-peak MET ^3^ level achieved on graded treadmill test	Risk of dementia-related mortality over an average follow-up period of 17 years	Individuals in the top and highest CRF tertile had lower risk for dementia-related mortality. The relative risk of dementia-related mortality decreased 14% for each 1 MET increase in fitness.
topton *et al.* 2008 [27]	7595	“High” *vs.* “Low/No” exercise based on response to two questions on a self-administered questionnaire	Cognitive decline based on performance on Modified Mini-Mental State Examination (mMMSE)	High exercisers showed less cognitive decline (3.1 *vs.* 5.5 pts on mMMSE over 5 years) when compared to low/no exercisers. Higher levels of exercise were associated with a lower risk of cognitive decline (10.3% *vs.* 15.8%) and a greater probability of cognitive improvement or stability (89.7 *vs.* 84.2%).
Yaffe *et al.* 2001 [28]	5925	Physical activity level measured by self-reported number of blocks walked or flights of stairs ascended per day and by the Paffenbarger Scale via interview	Cognitive decline (≥3 point decrease on mMMSE) at 6 and 8-year follow-ups	Odds of developing cognitive decline were 37% lower in the higher quartile of blocks walked (odds ratio, OR = 0.63) and 35% lower in the highest quartile of kcal expended (OR = 0.65).
Andel *et al.* 2008 [29]	264 dementia cases (2870 controls); 90 AD-discordant twin pairs	Self-reported physical activity	Risk for dementia development	Light exercise was associated with a reduced odds ratio of dementia (all-cause and Alzheimer’s) in case-control analyses. There was a non-significant reduction in odds ratio of dementia with higher activity levels in twin analyses.
Etgen *et al.* 2010 [30]	3903	Physical activity level (no, moderate, or high activity) based on self-reported activities	Cognitive performance measured using the 6CIT (higher score indicates more cognitive impairment) at baseline and 2-year follow-up	At baseline, 6CIT ^4^ scores were higher in no activity group compared with moderate and high activity. Cognitive impairment was more prevalent in the no activity (21.4%) compared to moderate (10.5%) and high (7.3%) activity groups. Moderate and high activity groups had reduced risk for cognitive impairment. No activity group had greater incidence of new cognitive impairment over 2 years compared to active groups.
topton *et al.* 2010 [31]	9344	Self-reported physical activity in teenage years, age 30, age 50, and late life (over 65). Classified as either inactive or inactive	Cognitive impairment determined by mMMSE score (impairment = score at least 1.5 standard deviation below the mean)	Physically active women at each age were less likely to have cognitive impairment in late life. Teenage physical activity status was most strongly related with reduced odds of late-life cognitive impairment.
Landi *et al.* 2007 [32]	364	Self-reported physical activity on questionnaire item related to frequency of high and light physical activity	Cognitive performance (Cognitive Performance Scale)	Those with a history of high-intensity physical activity had improved cognitive performance regardless of the age at which it was performed.
Tierney *et al.* 2010 [33]	90	Self-reported physical activity between high school and menopause	Postmenopausal cognitive performance (scores derived from a series of cognitive tests)	A positive relationship existed between moderate intensity activities and cognitive performance. A negative relationship existed between strenuous physical activities and cognitive performance.
Winker *et al.* 2010 [34]	114	Elderly marathon runners were compared to inactive controls	Cognitive performance (Vienna Neurophysiological Test Battery and CERAD ^5^ test battery)	Marathoners performed better in only one cognitive task (Five Point Test).
Arntzen *et al.* 2011 [35]	5033	Self-reported PA ^6^-classified as active or inactive based on 2 questionnaire items	Cognitive performance at 7-year follow-up	PA was associated with better cognitive performance in women, but not men.
Larson *et al.* 2006 [37]	1740	Self-reported physical activity (classified as physically active if they exercised at least 3 times per week)	Change in cognitive performance (using CASI ^7^) and incidence of dementia at biennial assessments over 6 years	Regular exercisers had a lower incidence rate of dementia (13.0 *vs.* 19.7 *versus* 1000 person-years), a higher probability of being dementia-free, and a lower age- and sex-adjusted risk of dementia compared to those exercising less than 3 times per week.
topton *et al.* 2011 [38]	197	Activity energy expenditure (AEE, measured using doubly labeled water)	Incidence of cognitive decline (based on mMMSE) over ~5 year follow-up	Levels of AEE were strongly associated with the likelihood of incident cognitive impairment (1.5% in highest tertile, 4.5% in the top, and 16.9% in the lowest). The top and highest tertiles were less likely to have incident cognitive impairment than the lowest tertile based on odds ratio.
Weuve *et al.* 2004 [39]	7982	Self-reported physical activity from Nurse’s Health Study questionnaires	Baseline cognitive function (Telephone Interview for Cognitive Status) and decline in cognitive function over 2 years	Those with highest physical activity levels at baseline had 20% lower odds of cognitive impairment compared to lowest quintile. Higher levels of physical activity were associated with less decline in most measures of cognitive performance.
Erickson *et al.* 2009 [41]	165	Cardiorespiratory fitness (CRF; VO_2_ peak ^8^)	Hippocampal volume and spatial memory task	CRF was a significant predictor of right and left hippocampal volume (HV), and modestly associated with performance on a memory task. Left HV was a significant partial mediator between fitness and spatial memory.
Erickson *et al.* 2012 [42]	137	Cardiorespiratory fitness (VO_2peak_)	Creatine and NAA ^9^ levels in the brain (MRS ^10^); cognitive performance (spatial memory and digit span task)	Age X fitness interaction indicated that aerobic fitness offset the age-related decline in NAA in the frontal cortex.
Honea *et al.* 2009 [43]	117	Cardiorespiratory fitness (VO_2peak_)	Regional brain volumes and associations with CRF in non-demented and mild AD patients	Atrophy was reported in the medial temporal, temporal, and parietal cortices in the mild AD group. CRF was associated with parietal and medial temporal volumes in mild AD patients but not non-demented adults. Apo ε4 genotype did not affect the relationship.

^1^ AD = Alzheimer’s-type dementia; ^2^ MCI = mild cognitive impairment; ^3^ MET = metabolic equivalent; ^4^ 6CIT = 6 Item Cognitive Impairment Test; ^5^ CERAD = Consortium to Establish a Registry for Alzheimer’s Disease; ^6^ PA = physical activity; ^7^ CASI = Cognitive Abilities Screening Instrument; ^8^ VO_2peak_ = peak oxygen consumption during graded exercise; ^9^ NAA = *n*-aceytlaspartate; ^10^ MRS = magnetic resonance spectrometry.

Although cross-sectional and prospective analyses provide data supporting a relationship between physical activity and cognitive function, they do not allow determination of a cause and effect relationship between exercise training and brain health. Interpretation of research findings is complicated by the questionable accuracy and reliability of self-reported physical activity, especially in the elderly population at risk for cognitive impairment. Other inherent faults in cross-sectional studies are the heterogeneous methods for measuring and quantifying physical activity, fitness level, and cognitive impairment, and the likeliness of a bidirectional relationship between cognitive impairment and physical activity. Investigations measuring cardiorespiratory fitness by maximal oxygen consumption (VO_2peak_) [26,41,42] have the advantage of presumably assessing physical activity level by more objective and reliable methods when compared to retrospective activity questionnaires or recall methods. The results of these investigations may therefore provide more reliable evidence of the relationship between physical activity and brain health. It is important to consider that although cardiorespiratory fitness logically relates to activity level, it is not necessarily reflective of activity levels, as cardiorespiratory fitness can be influenced by other factors such as chronic disease [44,45], especially in older, untrained populations. Moreover, the relationships reported may simply describe the likelihood that those with cognitive impairment and increased dementia risk are also those most likely to have physical limitations, chronic disease, or psychological issues that reduce their activity level and/or measured cardiorespiratory fitness. Furthermore, physical impairments that limit mobility may reduce access to social interaction with family and peers, which may influence cognitive function.

#### 3.2.2. Interventional Studies

Research designs employing exercise interventions (Table 2) provide the ability to examine a cause and effect relationship between exercise training and cognitive function. A recent meta-analysis of 29 studies involving aerobic exercise interventions reported modest but significant improvements in attention and processing speed, executive function, and memory in exercise-trained subjects [46]. Improvements in cognitive performance have been reported after supervised [47,48] and non-supervised [49] exercise interventions. In the latter, the exercise group, which was unsupervised but encouraged to increase their current physical activity level by 150 min per week for 18 months, improved performance on delayed recall and on the cognitive section of the Alzheimer Disease Assessment Scale (ADAS-cog) [49]. Improvements in the ADAS-cog and delayed recall occurred in the whole exercise group and also among those exercisers with MCI at baseline. In contrast, cognitive performance did not improve in previously sedentary, cognitively normal elderly subjects after an exercise intervention primarily involving walking for 150 min per week, although improvements in cognitive performance were correlated with improvements in physical performance [50]. 

**Table 2:** Summary of interventional studies that examined physical activity, fitness, and brain function. (**A**) includes studies that examined brain volumes; (**B**) includes studies that examined only cognitive function.

**(A)**						
**Study**	**Intervention/Subjects**	**Cognitive Function**		**Left Hippocampal volume**	**Right Hippocampal Volume**	
Erickson *et al.* 2011 [51] (*n* = 120)	1 year: Walking 3 days per week *vs.* stretching/toning control group	Both groups improved spatial memory task performance.		Increased 2.12% in exercise group. Decreased 1.4% in the control group.	Increased 1.97% in exercise group. Decreased 1.43% in control group.	
	Elderly adults without dementia (age 55–80)			Greater fitness improvements were associated (*r* = 0.37) with greater changes in hippocampal volume. Higher fitness level at baseline was associated with less hippocampal volume loss in the control group.		
Colcombe *et al.* 2006 [52] (*n* = 59)	6 months: aerobic walking 3 days per week *vs.* stretching/toning control group and non-exercising young controls	Not assessed		The aerobic exercise group showed an increase in gray matter (mainly frontal cortex) and white matter (anterior white matter tracts). Subjects in the aerobic training group had an average relative risk reduction for brain volume loss of 42.1%, 33.7%, 27.2%, and 27.3% in the anterior cingulate cortex, right superior temporal gyrus, right middle frontal gyrus, and anterior white matter clusters. The non-exercising young control group showed no change in brain volume.		
	Older adults (age 60–79) and group of young controls (age 18–30)					
Pajonk *et al.* 2010 [53] (*n* = 24)	3 months: cycling for 30 min on 3 days per week *vs.* non-exercise group that performed table tennis and exercise group of normal subjects	Memory improved in the schizophrenic exercise group more than the non-exercise group and the normal control group.		Hippocampal volume increased by approximately 14% in the combined exercise group: 12% increase in the schizophrenic group and 16% increase in the healthy control group. The change in relative hippocampal volume was related to the change in aerobic fitness in exercised schizophrenic and healthy control groups.		
	Schizophrenic individuals and healthy, normal controls (age 20–51)					
Parker *et al.* 2011 [54] (*n* = 13)	10 weeks: 3 days per week aerobic activity. No control group	Some improvements on computerized figural memory task		No significant change	No significant change	
	Healthy men and women (age 23–45)			Change in aerobic fitness was correlated with change in right (*r*^2^ = 0.31) and left hippocampal volume (*r*^2^ = 0.41).		
Liu-Ambrose *et al.* 2010 [55] (*n* = 155)	1 year: Full-body RT ^1^ on 1 or 2 days per week *vs.* Thai Chi and balance exercise control group	Stroop test performance improved by 12.6% and 10.9% in the 1/week and 2/week RT groups (0.5% decline in the control group)		Both RT groups showed a decrease in whole brain volume (−0.02% and −0.04% at 6 months; −0.43% and −0.32% at 1 year) with no change in control group		
	Women aged 65–75 without dementia					
** (B)**						
**Study**	**Intervention/Subjects**		**Cognitive Function**			
Baker *et al.* 2010 [46] (*n* = 33)	6 months: aerobic exercise 4 days per week *vs.* stretching control group		Executive function (multitasking, cognitive flexibility, information processing efficiency, and selective attention), but not short-term memory, improved in the exercise group compared to the control group.			
	Sedentary males and females (age 55–85) diagnosed with amnestic MCI					
Baker *et al.* 2010 [47] (*n* = 28)	6 months: aerobic exercise 4 days per week *vs.* stretching control group		Executive function, but not short-term memory, improved in the exercise group compared to the control group.			
	Adults (age 56–83) with impaired glucose tolerance					
Lautenschlager *et al.* 2008 [48] (*n* = 170)	18 months: Active group increased aerobic activity to 150 min per week (three 50 min sessions per week) *vs.* control group that did not		Subjects in the active group improved ADAS-cog and delayed recall scores more than the control group after 18 months.			
	Men and women over age 50 (102 with MCI ^2^)					
Williamson *et al.* 2009 [49] (*n* = 102)	1 year: physical activity intervention (primarily walking) or health education program		There were no differences in cognitive scores between groups after the intervention. Cognitive performance was correlated with changes in physical performance.			
	Sedentary elderly individuals without dementia (age 70-89)					
Cassilhas *et al.* 2007 [56] (*n* = 62)	24 weeks: full-body RT 3 days week at moderate-intensity (50% 1-RM ^3^) or high-intensity (80% 1-RM)		Both RT groups improved neurophysiological test performance when compared to the control group.			
	Sedentary males age 65-75 years					
Perrig-Chiello *et al.* 1998 [57] (*n* = 46)	8 weeks: full-body RT 1 day per week *vs.* control group		Improvements in the training group were seen in free recall (delayed) and recognition (immediate and delayed).			
	Men and women (age 65 to 95)					
Kimura *et al.* 2010 [58] (*n* = 119)	12 weeks: Progressive RT 3 days per week beginning at a 10-RM load		Executive function test performance did not change in either group.			
	Men and women (over 65 years)					

^1^ RT = resistance training; ^2^ MCI = mild cognitive impairment; ^3^ 1-RM = 1-repetition maximum.

Improvements in cognitive function have also been reported from supervised aerobic training interventions. Previously sedentary older adults with mild amnestic cognitive impairment at baseline improved performance on a series of tasks related to executive processing, but not short-term memory, after 6 months of aerobic training (4 days per week at 75% to 85% heart rate reserve) [48]. The control group that performed stretching and balance exercises showed no improvements in cognitive function. The intervention successfully improved cardiorespiratory fitness measured by maximal oxygen consumption (VO_2peak_) in the training group (+11%) compared to the control group (−7%). Interestingly, the treatment effect was larger in women for several executive function tests implying gender differences may exist in the response to exercise training. The same research group reported improved executive function, but not short-term memory performance in older, cognitively normal adults with impaired glucose tolerance relative to a stretching control group after a 6-month aerobic training intervention [47]. Comparatively, spatial memory improved in cognitively normal, elderly men and women after 1 year of aerobic exercise training, however, similar improvements were reported in the control group that performed only stretching exercises. Higher aerobic fitness level was correlated with spatial memory performance at baseline and at the end of the study for the combined sample, but fitness improvements were not related to improvements in memory. Baseline fitness levels may therefore be an important factor to consider when designing exercise intervention studies [51]. Findings from cross-sectional and experimental analyses support the benefits of exercise training for improving and maintaining cognitive function as indicated by improved performance on tests of memory task and executive function, and reduced risk of cognitive decline and dementia-related mortality in fitter and more active individuals. Problems with available interventional studies include the use of different tests for cognitive function, variability in the exercise training program, and an inability to examine the effects of lifelong activity patterns on dementia risk and cognitive function. 

## 4. Exercise and Neurobiological Changes

### 4.1. Animal Studies

Several studies in animals have examined changes in brain physiology in response to physical activity. In mice, expression of brain-derived neurotrophic factor (BDNF) increased in the hippocampus, and hippocampus and striatum, after level and downhill running, respectively [21]. Others have reported increased mRNA expression of neurotrophic factors [23,59], enhanced signaling in neurogenerative pathways [21], increased expression of neurotransmitters [20,59], and favorable changes in energy metabolism and neurodegenerative markers [60] in the hippocampus of exercised mice. Furthermore, aerobic exercise reduced inflammatory cytokines and oxidative stress [61,62], and increased antioxidant capacity [23] in mice hippocampi. Treadmill exercise also prevented impairment in cognitive function and deleterious neurochemical changes (e.g., oxidative stress, reduced neurotrophic factor levels, neurodegeneration) induced by proline or streptozotocin administration in rats [63,64]. These findings indicate that exercise improves possible mediators of age- and stress-related neurodegeneration, and suggest that anti-inflammatory and antioxidant effects as well as neurochemical alterations in the hippocampus and cortex could mediate putative changes in neurologic function induced by exercise in humans. 

Compelling findings from animal studies also suggest exercise training may prevent or reverse age-related changes in brain tissue associated with dementia [59,65,66]. Hippocampal cell proliferation was greater, but there were no differences in neurogenesis between physically active and sedentary transgenic mice predisposed to AD [67]. The findings were the same for physically active mice raised in an environment enriched with cognitive activities. In comparison, both cell proliferation and neurogenesis were enhanced in the dendate gyrus of old mice after 45 days of running when compared to sedentary counterparts [68]. Similarly, running exercise restored hippocampaal neurogenesis and dendritic remodeling after cortisone administration [22], and exercise during midlife reduced glial and vascular markers of aging in old mice [69]. Collectively, these findings suggest that chronic exercise may be protective against age-related changes in neurobiology, including altered expression of neurotrophic factors and neurotransmitters, and enhanced or maintained neuronal proliferation and maturation in the hippocampus. 

### 4.2. Human Studies

#### 4.2.1. Cross-Sectional and Prospective Studies

Cross-sectional and prospective human investigations suggest a relationship between fitness/physical activity level and brain health. In a prospective analysis of older adults, physical activity level at baseline (measured as self-reported number of blocks walked per week) predicted gray matter volume changes over 9 years [13]. In cross-sectional analyses, aerobic fitness (VO_2peak_) was associated with gray matter volume and white matter integrity in females with relapsing-remitting multiple sclerosis [70] and with parietal and medial temporal volume in early-stage AD patients [43]. Moreover, cardiorespiratory fitness, measured by VO_2peak_, was a significant predictor of right and left hippocampal volume, explaining 7.8% and 12.2% of right and left hippocampal volume, respectively in a group of older adults age 59 to 81 years [41]. Higher aerobic fitness was associated with greater right and left hippocampal volumes, but only left hippocampal volume was reported to be a significant partial mediator of the relationship between aerobic fitness and memory. In addition to hippocampal volume, physical fitness was related to brain levels of NAA [41]. Age is associated with a decline in NAA concentrations in the frontal cortex, but this may be offset by higher fitness levels [42]. NAA concentrations were similar between high and low fitness groups (based on VO_2peak_) in middle-age subjects (age 58–65), but concentrations were greater in fitter when compared to less fit older subjects (age 66–80). These cross-sectional investigations indicate a relationship between higher aerobic fitness level, larger hippocampal volume, and improved neuronal health, and suggest that improvements in cognitive function with aerobic activity may be mediated by neurophysiological and structural changes in the brain. 

#### 4.2.2. Interventional Studies

**Figure 2:**
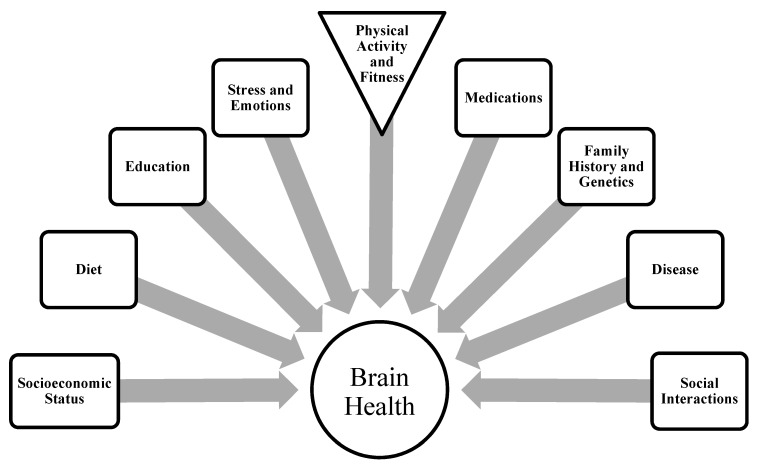
Schematic representing the numerous factors that modulate the relationship between brain function and physical activity.

Interventional studies indicate aerobic exercise training increases regional brain volumes. Regional gray and white matter volumes increased and relative risk of brain tissue loss decreased in older non-demented adults performing aerobic exercise training for 6 months when compared to a control group that performed stretching and toning exercises [52]. Hippocampal volume increased between approximately 2% to 16% in small samples of young, healthy subjects after aerobic exercise training [53,54]. In a large sample (*n* = 120) of cognitively normal older adults, left and right anterior hippocampal volumes increased after 1 year of aerobic exercise training (walking 4 days per week at 60%–75% HRR) [51]. Improvements were relative to a control group that performed toning and stretching exercises. However, the changes in left (+2.12% in aerobic *vs.* −1.40% in control) and right (+1.97% *vs.* −1.43%) hippocampal volumes were modest. In addition, the standard deviation for volume measurements was 3–4 fold larger than the reported mean difference (~0.15 cm^3^) between groups indicating that the treatment effect was not large and there may be considerable intra-individual variability in the response. The large variability in hippocamal volume measurements is likely attributable to the multitude of biological and environmental factors that influence human brain physiology (Figure 2). 

There appears to be a relationship between fitness improvements and hippocampal volume changes with exercise training. After 1 year of aerobic training, greater improvements in aerobic fitness (VO_2peak_) were moderately but significantly (*p* < 0.001) associated with greater changes in left (*r* = 0.37) and right (*r* = 0.40) hippocampal volumes. In addition, higher fitness level at baseline was associated with less loss of right anterior hippocampal volume in the control group that did not exercise [50]. Similarly, our group reported modest (*r*^2^ = 0.41) but significant (*p* = 0.02) associations between changes in cardiorespiratory fitness (VO_2peak_) and changes in left hippocampal volume after a 10-week aerobic exercise intervention (3 days per week for 40 min at a moderate intensity) in 13 healthy men and women (age 23–45) [54]. Right and left hippocampal volume did not change, which may be related to the heterogenous fitness improvements observed (change in VO_2peak _range: 0 to 22%). The relationship between changes in cardiorespiratory fitness and increased hippocampal volume suggest that a longer duration intervention more likely to induce more uniform and substantial improvements in fitness may increase hippocampal volume. Increased regional brain volumes in older adults occurred concomitant to significant improvements in aerobic fitness ranging from 8% to 16% [51,53]. Together these findings suggest that improvements in cardiorespiratory fitness may mediate the effects of exercise training on brain volume, and suggest that exercise interventions that result in greater fitness improvement will elicit greater changes in brain volume. However, such theories are speculative and conflict with two meta-analyses of cross-sectional and longitudinal studies examining physical activity and cognition, including a Cochrane review of 11 RCTs [71], which concluded that aerobic fitness changes did not mediate changes in cognitive performance (regional brain volumes not measured) [72].

#### 4.2.3. Regional Brain Activity

Regional brain activity, measured by changes in the blood oxygen level dependent (BOLD) signal intensity during functional MRI, is altered in those at risk for AD [73,74], in declining MCI [75,76], and in diagnosed AD [74]. The nature of these changes is not well understood, but there appears to be hyperactivity in the medial temporal lobe during cognitive stimulation in the early stages of MCI, prior to the onset of dementia, that is thought to be a consequence of reduced neuronal efficiency [74,75]. As cognitive impairment progresses, hippocampal activation is reduced and there is a compensatory increased in activity in other regions, including the parietal regions [74,76]. Higher fit and aerobically trained groups demonstrated increased prefrontal and parietal cortical activity and decreased activity in the anterior cingulate gyrus during a flanker task designed to elicit activation in the frontal and parietal lobes [77]. Greater activity in a large neural network associated with memory and learning, including the hippocampus, was associated with individual cardiorespiratory fitness level before and after a 6-month cycling program, but only in a group that also performed spatial navigation training [78]. Improvements in cardiorespiratory fitness with training correlated with increased activity in the frontal cortex, the cingulate gyrus, the insula, and the parahippocampal gyrus. Changes in regional brain activity may be related to the previously discussed structural and neurophysiological changes in the brain and may contribute to the cognitive benefits of exercise training. 

## 5. Resistance Training

Several studies have examined the effects of resistance or strength training on cognitive function. In rats, aerobic training (treadmill running) and resistance training (vertical ladder climbing) improved spatial memory task performance; however, training modalities were associated with divergent neurochemical changes in the hippocampus [79]. Improvements in cognitive function were reported in three randomized clinical trials (RCT) of elderly men and women after full-body resistance training programs lasting between 8 weeks and 1 year [55,56,57]. Improvements were independent of frequency (one or two sessions per week) [55] and intensity (moderate *versus* high-intensity) [56]. In contrast, 12 weeks of progressive resistance training (3 days per week) had no effect on executive cognitive function in another RCT of elderly subjects. Only one study assessed changes in brain volume, and reported a small reduction in whole brain volume after resistance training (−0.43 to −0.32%) [55]. These limited studies and their divergent results suggest that the relationship between chronic resistance training and brain function has not been adequately studied to draw conclusions. 

## 6. Conclusions and Directions for Future Research

Cross-sectional, prospective, and interventional studies provide promising, but not consistent, findings supporting cognitive benefits from regular exercise. Specifically, exercise training and physical activity may maintain or improve performance on hippocampus-related tasks via increased volume or neurophysiological changes. There are major deficiencies in our current understanding of the relationship between physical activity and brain function including the following:

### 6.1. How Does Exercise Benefit Brain Function in Special Populations at Risk for Accelerated Brain Tissue Atrophy and Dementia?

Certain diseases place individuals at an increased risk for the development of dementia and neurodegenerative changes. Studies performed in type 2 diabetics have reported increased risk for dementia [66,80], impaired cognitive performance [81], and accelerated whole brain and hippocampal atrophy [81,82,83,84,85] when compared to non-diabetics. Most of these studies included diabetics being treated with oral anti-diabetic agents and/or insulin therapy and did not exclude subjects with diabetic complications including cardiac disease, hypertension, and vascular disease. Consequently, there have been divergent findings regarding dementia risk and neurophysiological changes in diabetics, possibly because of disparities in diabetes duration, medical therapies used, presence of complications, and the degree of glycemic control within subjects groups [81,83,86]. 

There is some literature supporting an increased risk for dementia as well as decreased volume and/or altered shape of the hippocampus in depressed older adults [87,88,89,90], and particularly in those who experience depressive symptoms in late-life [91]. The effects appear to be more pronounced in the left hemisphere [90,92,93] and left hippocampal volume is inversely related to the rate of cognitive decline in older depressed adults [92,93]. Other conditions associated with accelerated neurodegeneration and cognitive decline include obesity [94,95,96,97], hypertension [85,98], and prior cerebral hypoperfusion injury [99,100,101,102]. In addition, cancer patients and survivors may be susceptible to cognitive decline and neurodegeneration due to the effects of chemo-therapy [103,104]. Investigations are needed into the effects of habitual physical activity level and structured exercise interventions on the rate and extent of cognitive decline and neurodegeneration in these vulnerable populations. 

### 6.2. How Does the Nature of the Exercise Intervention or Physical Activity Influence Cognitive Function and Brain Volume/Physiology?

Cross-sectional studies suggest those who maintain an active lifestyle better maintain cognitive function and brain tissue integrity [24,25,28,31,41,42,51]. Improved cognitive function and small changes in brain volume were reported after exercise interventions lasting between 6 months and 1 year [48,51,52]. It is unclear if this is a sufficient period of time to adequately assess neurobiological changes in response to exercise. Furthermore, it may be that maintaining an active lifestyle across the lifespan prevents or reduces some of the age-related changes in cognitive function and brain physiology rather than improving these factors per se. Longer-term exercise studies are necessary to determine if (a) exercise can reduce loss of brain tissue volume and cognitive decline with age and (b) long-term chronic exercise across the lifespan can maintain brain function and tissue integrity. In addition, it remains to be determined if activity initiated in late-life confers equivalent benefits to lifelong physical activity, and if the time point, or age, at which the intervention is initiated influences the effectiveness of the intervention. 

Other questions that remain include if a threshold “dose” of exercise is necessary to see changes in brain function, specifically does the intensity, duration, or frequency of the exercise session matter? Furthermore, for how long will exercise-induced improvements persist after cessation of exercise training or during periods of reduced physical activity? Improved performance on only 1 of 4 cognitive tests was maintained 1 year after cessation of a 12-month resistance training program [105], but further research is necessary to determine if aerobic exercise training leads to lasting improvements in brain function. Answering these questions requires a series of investigations comparing different levels of exercise intensity, duration, or frequency. Dose-response determination is complicated by seemingly limitless combinations of training variables that can be prescribed. Finally, the effects of exercise modality on brain function have not been adequately studied. It remains to be clarified if physical activities requiring higher levels of motor complexity or that require more memory function will differentially impact cognitive function or brain physiology. Thus, it is necessary to compare relatively simple motor tasks, such as walking, with activities that are more complex, such as dancing, game playing, and resistance training. 

### 6.3. How Does Physical Activity Interact with Certain Medications to Affect Brain Function and Physiology?

Certain medications may improve or impair cognitive performance and thus have synergistic or antagonist interactions with exercise training interventions. For example, some antiepileptic medications have been associated with improved (e.g., levetiracetam, tiagabine) or impaired (e.g., phenobarbital, primidone, carbamazepine, phenytoin, and topiramate) cognitive function [106]. There is also limited evidence of a relationship between anticholinergic medications and cognitive impairment [107]. Carriers of the ApoE epsilon-4 allele may be especially sensitive to cognitive effects of anticholinergic medications [108]. Conflicting reports exist regarding the relationship of statin usage, cognitive function, and dementia risk [109]. In a few studies and in select few patient reports, impaired memory and cognitive performance have been reported approximately 2 months after statin therapy initiation [110,111,112]. Others have reported no change [113] or improved [114,115,116] cognitive function with statin therapy. There are promising findings from animal and human studies that statins may prevent or attenuate pathological neural and vascular changes associated with vascular and Alzheimer’s dementia [117,118,119]; for reviews see [120,121,122]. The interaction between pharmacological therapy and physical activity in influencing neurobiology and cognitive function is a relatively unexplored area that deservers further attention. 

In conclusion, current research suggests that active individuals and those prescribed structured exercise regimes demonstrate improved cognitive function and reduced brain atrophy and/or neurodegeneration, particulary in the hippocampus. The relationship between physical activity and brain function is complicated by a multitude of modifying factors, including stress, education, socioeconomic status, medications, diseases, cardiovascular risk factors, and other health behaviors that are nearly impossible to control in human studies (Figure 2). The potential for exercise training to delay or attenuate age-related decrements in cognitive performance and neurobiology is an important field of study because of the possible benefit to the health and independence of a growing elderly population and to the larger social and economic environment. Directions for future research include targeting vulnerable clinical populations, optimizing exercise training variables, and examining the synergistic relationship between pharmacological and non-pharmacological therapies. 
